# Response: Commentary: The effects of acute stress on core executive functions: A meta-analysis and comparison with cortisol

**DOI:** 10.3389/fpsyg.2017.02090

**Published:** 2017-12-08

**Authors:** Grant S. Shields

**Affiliations:** Psychology, University of California, Davis, Davis, CA, United States

**Keywords:** acute stress, executive function, response inhibition, selective attention, sustained attention, interference control, commentary, meta-analysis

Dang (2017) commentedon our recent meta-analysis (Shields et al., 2016) that reviewed the effects of acute stress on executive functions. A conclusion of our review was that stress impaired “cognitive inhibition” (i.e., selectively attending to or ignoring information) but enhanced “response inhibition” (i.e., inhibiting a prepotent response). Dang argued that many specific tasks^1^should have been classified differently than they were in the meta-analysis and, thus, the effects of stress on inhibition are still pending. However, our task classification in the meta-analysis was not arbitrary; rather, it was based on previous research. Moreover, although the new classification proposed by Dang may appear reasonable in some ways, it is not strongly supported by the existing literature.

One classification with which Dang takes issue is the forward span task, arguing that it should be classified as working memory rather than inhibition. Performance on forward span tasks is indeed underpinned in part by cognitive processes associated with performance on working memory tasks, such as storage processes (Baddeley, 2000). However, storage processes are not executive functions, and performance on forward span tasks is not a measure of the executive component in working memory (Baddeley, 2000, 2012; Miyake et al., 2000; Conway et al., 2002; Repovš and Baddeley, 2006; Diamond, 2013). Indeed, neuropsychological research has established that performance on forward span tasks is underpinned not by working memory, but cognitive inhibition (i.e., selective/sustained attention): “What Digits Forward measures is more closely related to the efficiency of attention (i.e., freedom from distractibility) than to what is commonly thought of as memory” (Lezak, 2004, p. 353; see also Gold et al., 1997; Gómez-Pérez and Ostrosky-Solís, 2006; Tian et al., 2015). The same is true for tasks measuring simple or choice reaction time: the only executive function required for performance on them is cognitive inhibition in the form of selective and sustained attention (Stuss et al., 1989; Bonnelle et al., 2011). Thus, performance on these tasks is underpinned primarily by cognitive inhibition, justifying our classification of them as cognitive inhibition tasks.

Classifying the above tasks as cognitive inhibition might lead one to argue that theoretically all tasks could be classified as cognitive inhibition, as performance on any given cognitive task is of course dependent on attending to the presented stimuli. However, we classified tasks according to the cognitive function the task primarily depended upon for performance. Therefore, we did not include tasks that primarily relied on another cognitive function for performance (e.g., reading comprehension) in our analyses.

Dang also contends that the sustained attention to response task (SART) is a response inhibition task, not a cognitive inhibition task. The reason for this contention is that the SART has a similar structure to the go/no-go (a response inhibition task; e.g., Rubia et al., 2001). However, the SART differs from a typical go/no-go in a number of ways. For example, in classic go/no-go tasks, there are two stimuli presented. A response is made to one of these stimuli, which is presented in a large proportion of trials (e.g., 75%), whereas a response is withheld to the other stimulus, which is presented in the remainder of the trials. However, in the SART, nine stimuli are presented, each with an equal proportion of occurrence (11%), and responses are withheld for only one of these nine items (Robertson et al., 1997).

Importantly, the particular task characteristics of the SART lead to it correlating with other sustained attention (i.e., cognitive inhibition) tasks but not with classic response inhibition measures (Robertson et al., 1997; see also Manly et al., 1999). Moreover, Christoff et al. (2009) presented both behavioral and neural evidence that even errors of commission on the SART reflect lapses in sustained attention—not failures of response inhibition. Therefore, evidence suggests that the SART depends primarily on cognitive inhibition.

Additional evidence for classifying the SART as cognitive inhibition comes from a reanalysis of the data from the meta-analysis itself. In particular, it is possible to test whether studies that used the SART (*m* = 4) differed from studies that used response inhibition tasks (*m* = 6) or from studies that used other cognitive inhibition tasks (*m* = 13). In these analyses, the effect of stress on SART performance (*g*^+^ = −0.290) was significantly different from the effect of stress on response inhibition performance (*g*^+^ = 0.295), *B* = −0.585, *t*_(6.4)_ = −3.57, *p* = 0.011, but the effect of stress on SART performance did not significantly differ from the effect of stress on other cognitive inhibition tasks (*g*^+^ = −0.182), *B* = −0.108, *t*_(5.1)_ = −0.67, *p* = 0.531. Thus, the effects of stress on the SART were quite similar to the effects of stress on other cognitive inhibition tasks but quite different from the effects of stress on response inhibition tasks, corroborating the findings of prior research indicating that the SART is a cognitive inhibition task.

A final point contested by Dang was classifying certain newly developed tasks and complex emotional interference tasks as cognitive inhibition tasks. Dang states that because Sänger et al. (2014) used a novel task to assess cognitive inhibition, the validity of the task is unclear. However, the reason behind this claim is unclear, since Sänger et al. validated the task as a cognitive inhibition task with ERPs. Similarly, Dang states that the task used by Giles et al. (2015) should be classified as working memory. However, this task was validated in prior literature (e.g., Erk et al., 2007) to primarily assess “attentional control and the ability to regulate emotion” (Giles et al., 2015, p. 12).

Dang's conclusion is that stress effects on inhibition are still pending. I agree. The meta-analysis included many heterogeneous paradigms, and the number of studies that examined response inhibition in it was small. Nonetheless, despite these limitations, the task classifications—and therefore, inferences—we made were determined by and are in agreement with prior research, whereas the classifications suggested by Dang were and are not. Future work should therefore be careful to follow established classifications for executive function tasks (e.g., Kornblum et al., 1990; Miyake et al., 2000; Nigg, 2000; Diamond, 2013).

## Author contributions

The author confirms being the sole contributor of this work and approved it for publication.

### Conflict of interest statement

The author declares that the research was conducted in the absence of any commercial or financial relationships that could be construed as a potential conflict of interest.

## Footnotes

^1^Technically, tasks are not measures of executive functions—outcome measures derived from them are. I use “task” hereafter for brevity.
